# An organizational working time reduction and its impact on three domains of mental well-being of employees: a panel study

**DOI:** 10.1186/s12889-024-19161-x

**Published:** 2024-06-28

**Authors:** Francisca Mullens, Ilse Laurijssen

**Affiliations:** 1 Research Group BRISPO, Vrije Universiteit Brussel, Pleinlaan 2, Brussel, 1050 Belgium; 2 Research Group BRIO, Vrije Universiteit Brussel, Pleinlaan 2, Brussel, 1050 Belgium

**Keywords:** Well-being, Working hours, Shorter workweek, Time-use, Work-family conflict, Working time reduction

## Abstract

**Background:**

Work hours are an important aspect of one’s job and these in turn have the potential to impact people’s well-being. Much research investigating the link between working hours and well-being uses cross-sectional data. Longitudinal studies, especially those studying the same subjects changing their working time, can study the impact of work time more clearly. Using panel data, this study aims to explore the impact of a reduction in working time on three domains of well-being: general well-being, job-related well-being (positive work experience) and work-family well-being (work-family conflict). In addition, our study offers insights into the role of concomitant changes in work and private circumstances of employees as we investigate whether the impact of shorter working hours for well-being is mediated by changes in the participants’ and circumstances related to paid and unpaid work resources.

**Method:**

An organization of about 60 (female) employees trialed a shorter workweek for one calendar year in 2019. All full-time employees reduced their hours. The part-time working employees can be used as a control group. Panel data (survey and time-use diary data) of a 30-h workweek trial in Belgium was collected in four waves over two years in a pre- and post-intervention design. Change over time (waves) was analyzed through multilevel growth models.

**Result:**

A decrease in work-family conflict was observed during the shorter workweek. Part of this decrease is explained by concomitant changes in work and private circumstances, such as sufficiency in free time, schedule control, and satisfaction with work pressure. Positive work experience and general well-being tend to have decreased during the shorter workweek, although this could partly be explained by other organizational changes and not by the reduction in working hours per se. Schedule control helped suppress these somewhat negative effects of organizational changes on positive work experience.

**Conclusion:**

Reduced working hours have the largest and most positive impact on work-family conflict. The feeling of having enough leisure time contributes to this increased well-being. Especially for women, who were the majority in this study, a reduction in working time might be beneficial as they often bear more responsibility for household work and care tasks. Next to the duration of working time, schedule control/autonomy has an important impact on well-being.

**Supplementary Information:**

The online version contains supplementary material available at 10.1186/s12889-024-19161-x.

## Introduction

Work hours are an important aspect of one’s job and these in turn have the potential to impact people’s well-being. Reductions in working time have often been introduced with the (partial) aim of improving workers’ well-being [21]. However, most research on working hours and their impact on well-being uses cross-sectional data, comparing people with different amounts of working hours. While some of these studies find lower well-being when working longer hours (e.g., [4, 27]), review studies show rather weak or inconsistent support for the negative association between long hours (and overtime) and mental health [16, 48]. The inconsistent findings could be due to different conceptualizations of mental health but could also be related to the cross-sectional design of these studies in which other work-related and personal conditions might impact subjective well-being. Longitudinal studies into the well-being of the same people over time might help to overcome the latter issue, and in particular cases studying the consequences of collective work time reductions. Even though trials and large-scale reductions in the last decades are still rather scarce, the evidence available shows a positive impact of work time reduction on workers’ well-being (e.g., [18, 19, 31, 46]). Adding to that body of knowledge, we report on results from a 30-h workweek work time reduction trial conducted in a Belgian organization in 2019. A strength of our study is the dynamic perspective, as we have measured (the almost exclusively female) employees’ well-being both before and after the introduction of the working time reduction, and can compare changes in well-being between employees whose work time was reduced (more) and others whose work time did not reduce or reduced less. We look into the impact of this case of collective working time reduction on three domains of mental well-being, namely employees’ feelings of general (or context-free) well-being, job-related well-being and work-family well-being, corresponding to the three domains of well-being discerned in Fox’s et al. review study [14] on the impact of workplace interventions on worker well-being. In addition, our study offers insights into the role of concomitant changes in work and private circumstances of employees as we investigate whether the impact of shorter working hours for well-being is mediated by changes in time use, circumstances related to unpaid work, and (paid) work resources of respondents.

## Background: the impact of work time reduction

Working time reduction might yield a ‘triple dividend’, impacting social, economic, and environmental dimensions [3, 18]. These dimensions correspond to the three large motivations behind working time reductions historically [21]. While the environmental and economic dimensions primarily entail collective and societal impacts (although individual/employee productivity can also be part of the economic dimension), the social dividend encompasses mostly individual-level implications such as well-being. This paper investigates how a reduced workweek can contribute to individuals’ well-being.

There has been little research on the impact of working hours on mental health or well-being that uses a longitudinal design. In one example, using longitudinal data, Gash et al. [17] found that women who reduced their working hours while staying in the same job felt happier over time. The authors speculate that most of these women voluntarily reduced their hours from full-time to part-time and this might help explain the positive results. Their hours are more in line with their preferences and this work hours fit is known to impact well-being (e.g., [41]). When women switch from full-time to part-time this is often an individual choice based on gendered preferences. These working time preferences ‘are usually compromises between what is desirable and what is feasible’ ([8], p. 16). The individual’s decision to reduce working hours is influenced by their conditions, (gendered) norms and preferences. However, collective reductions are less dependent on these individual factors. By studying the effects of changing work hours in collective reductions on a national or organizational level, researchers can separate and analyze them more clearly. This also offers a more promising context to study the impact of working less on mental well-being and some examples of this type of research are already available. In Korea for example, Rudolf [37] studied the impact of a national working time reduction on overall life and job satisfaction. He concluded that the policy did not improve overall satisfaction with one’s life and job [37]. The author suggests that this could be due to an intensification of work and downward adjustments in leave [37]. Other country-wide reductions in working time such as those in France in the early 2000s (from 39 to 35 h) and Portugal in 1997 (from 44 to 40 h) did show an improvement in workers’ well-being: using the European Community Household Panel, Lepinteur [31] found that the reforms increased both the job satisfaction and leisure satisfaction of workers [31]. Investigating the impact in France, Fagnani and Letablier [12] find that parents of young children did have positive opinions on the impact of the shorter working hours, however, the reduction of hours was not large enough for all parents to improve work-family balance. The authors conclude that the context of these changes (the size of the organization, the form of reduction, e.g., weekly vs yearly reductions, etc.) should be considered.

Next to research on national work hours reductions, there are a few smaller-scale trials with reductions on an organizational level that have been studied. A review study [46] on 7 studies based in Northern Europe mainly in health care sectors, looked into the impact of organizational reductions in working hours on health outcomes. The study concludes that working time reduction improves working-life quality. Only three cases also evaluated the effect on the quality of life outside of work but did not find improvements in this area. However, [5] reported a decrease in work intrusion on private life, while [47] did not find any significant effects for work-home interference. Hanbury’s et al. [18] recent systematic review study showed that the strongest evidence and most pronounced effects of working time reduction can be found within the social dividend, meaning that it has the strongest impact on personal health and well-being (life satisfaction, quality of life, work-family conflict, etc.) of the employees. Similar findings were reported in the scoping review of Karhula et al. [26]. Other case studies, that were not included in either of the above-discussed review studies, such as the recent experiments in Iceland and the UK, preliminarily also show positive impacts on job-related well-being: increases in job satisfaction, feeling well at work, and job motivation [19, 42].

Although review studies generally point to a rather positive impact of collective working time reductions on employees’ well-being, the overall evidence presented by the above-discussed studies seems somewhat inconsistent. This inconsistency might be due to deviations in the definition and measurement of (long) working hours (e.g., [20] as well as well-being [9]). Inconsistent findings can also be related to different conceptualizations and contexts in which the implementations took place [18]. These contexts relate to how the reduction is implemented (form), the extent and the level of implementation, what other changes took place (such as some organizations not reducing the workload, resulting in increased work intensity) (e.g., [37]), the work conditions and wage compensation, but also the broader (national) social and gender context [1]. We aim to emphasize in this study that it is not just concomitant changes in work and work context that are significant to changes in well-being in collective reduction of work hours, but also the concomitant changes that happen in the private realm and the household context related to this reduction.

### Changes in work context

Working time reductions might change work conditions or the work context, which can in turn also affect well-being. Anttila et al. [1] found different impacts by socio-economic status: only manual and lower-level white-collar employees experienced a reduction in work-family conflict, while upper-level white-collar employees did not. Similarly, Hanbury et al. [18] found smaller reductions in work-life conflict for upper-level white-collar workers. The authors relate these differences to upper-level white-collar employees having to do the same tasks in less time and having less autonomy over their work timing than before, while on the other hand, blue-collar workers might in general have fewer other resources to cope with work stressors and therefore a reduction in work time might have a larger impact. Other research has shown that perceived control over time (time autonomy) can act as a moderator between working hours and occupational stress (e.g., [24]). Occupational stress might also be impacted by work intensity or work pressure. Especially when a reduction in working hours is not accompanied by a reduction in workload, (imposed) work intensity/pressure might rise [9, 29]. The socio-economic aspect might also be important in our Belgian case study in that almost all workers (80%) are highly educated and are categorized as ‘knowledge workers’. These workers also have a lot of temporal autonomy over their work. We thus expect that changes in both work pressure and schedule control/autonomy over work will affect job-related well-being and general well-being as well.

### Changes in the private realm and time use

How the extra time off is spent might also affect the experience of a working time reduction [46], yet this aspect is often absent from research. Having time for activities other than work might be of importance when it comes to workers’ well-being, as it makes room for recovery. Especially time spent on non-work activities [45], free time activities [28, 30] and socialization activities and sports [15] are associated with higher levels of happiness and well-being. On the other hand, spending more time on childcare, spousal care activities and housework was found to be detrimental to mental health, especially for women [32]. Rather than actual time spent on housework, Thomas et al. [43] found that it was the perceived unfairness of the division of household labor that was associated with reduced health and well-being for women. Differential effects of work hours on well-being for women and men, such as more negative impacts of long hours on women’s depressive symptoms [49] or a better work-life balance for part-time working women [6] can be explained by gendered roles and expectations, with women more responsible for the family, while also taking up the work role (double burden/shift) (e.g., [7, 22]). Anttila et al. [1] found that shorter daily hours reduced time-based work-family conflict for women, but only for those with children. Especially those well-being indicators related to the combination of both the family and the work role might thus be affected in women. Nevertheless, how the freed-up time is spent, might also impact more general well-being.

## The Femma Wereldvrouwen case of work time reduction

This study reports results from a shorter workweek trial based in Belgium in 2019. As a non-profit women’s organization in the socio-cultural work sector, Femma Wereldvrouwen also supports a collective working time reduction on a societal level to reduce the inequalities in time use between women and men and to improve the often difficult combination of both paid and unpaid work for individual employees. Femma Wereldvrouwen opted to trial a shorter workweek themselves to see what the impact would be on their employees. For 12 months about 60 employees (varying over the years due to end of contracts and retirements) trialed a 30-h work week. All full-time employees reduced their working hours to 30 per week and retained their full-time salary. A normal full-time workweek before the 30-h workweek at Femma Wereldvrouwen comprised 36 h, yet some worked a full-time week of only 34 or 32 h. This already existing small reduction is not part of the trial and was based on age and agreed upon on a sectorial level. The age-based reduction is a union-acquired right for all end-of-career employees in specific sectors in Belgium and should be understood in the context of workable work. All employees over 50 could work 34 h full-time and those over 55 could work 32 h full-time. These groups thus reduced their working hours respectively by 4 or 2 h to 30/week in the 30-h workweek trial. Next to full-time workers, Femma Wereldvrouwen also had a group of part-time workers who did not reduce their hours but did receive a proportional wage increase. The part-time workers working 28 h/week before the trial had the option to increase their work hours to 30 and receive full-time pay. The employees at Femma Wereldvrouwen are fairly homogeneous: all but one were women and most (80%) were highly educated (with a college or university degree).

Organizational commitment and change to accommodate a shorter working week together with employee consultation seem important for successful implementation, especially regarding positive impacts on workers’ well-being (e.g.,[11, 25]). Aware of this importance and committed to not increasing employees’ workload, Femma Wereldvrouwen sought support from organizational change consultants and installed a work group of employees who were co-responsible for the organizational changes in the run-up to the 30-h workweek. As part of this reorganizational plan, new employees were hired, and some tasks were outsourced during the trial to relieve pressure on employees. In addition, new self-managing teams were introduced several months before the start of the shorter workweek. Self-management, however, was not easy for all teams, and some struggled the first half year/year. A few teams (comprising 33% of employees) in particular deviated from other teams and management had to intervene in how they worked together and even started the resignation procedure for those employees who could not cope with the change. This affected the work atmosphere in these teams and might have impacted other teams as well.

### Aim of our study and hypotheses

Using data on the effects of this collective reduction of working time trial, this study adds to the scarce evidence on the impact of collectively introduced shorter workweeks on mental well-being among female knowledge workers. This study will investigate changes in the three important domains of mental well-being: namely general (context-free) well-being, job-related well-being, and work-family well-being [14]. Our first hypothesis relates to work-family well-being. We hypothesize that a working time reduction will improve work-family well-being (H1a) because the burden of paid work is lowered in terms of duration which decreases the dual burden many women experience. Yet we expect that this might be mediated by how the extra time off is used. Extra time spent on leisure might be more beneficial (H1b). To the best of the authors’ knowledge, this will be the first paper including time-use data in the analysis of the impact of a collective shorter work week on mental well-being. Our second hypothesis relates to job-related well-being: following recent research on shorter workweek experiments discussed above, we expect job-related well-being to improve during the shorter workweek (H2a). However, changes in schedule control or work pressure will probably also affect this domain of well-being (H2b). Lastly, for our third hypothesis, we expect general well-being (like life satisfaction in Lepinteur’s study, [31]) to improve in the shorter workweek (H3a), which is also a consequence of the increase in job-related well-being and work-family well-being (H3b).

## Data & methods

### Data

Research unit TOR at the BRISPO research group of the VUB was approached for its expertise in the study of time use and asked to study the impact of Femma Wereldvrouwen’s shorter workweek trial. Although some alignment was necessary, TOR conducted independent research and Femma Wereldvrouwen was not involved in the hypotheses, data analyses or discussion of results. We set up a panel study consisting of five waves of data collection both before, during, and after the 30-h workweek trial. In each wave, a 7-day time-use diary and survey (pre- and post-diary) data were collected from all employees. The first two waves took place in March and October of 2018, the year before the trial. These are the pre-measurements. The third and fourth waves took place during the trial, in March and October 2019. The last wave took place in March 2020, some months after the end of the trial. Unfortunately, the first Covid-19 lockdown started in the middle of March in Belgium and most of the employees were still in the process of keeping their time-use diary when everyone was told to stay home. We cannot compare this wave to earlier waves. We will thus only use the pre- and during the shortened workweek (also called pre- and post-intervention) measurements of 2018 and 2019 to study the impact of the working time reduction. Table 1 shows the total number of employees invited and participating in all stages of the included waves. In the pre-diary survey, respondents were asked about their work hours, socio-demographic background variables, their workplace experiences, experienced time use, and schedule control. In the time-use diary, respondents registered in detail all of their activities with timings during seven days. The post-diary survey included questions on their filled-in diary week (was this a normal week etc.), transport, the division of unpaid work, and questions on well-being. Table A1 in the appendix lists for each dependent and independent variable used in this article with which instrument the data were collected. All data was collected through the MOTUS software [35].

**Table 1:** Number of employees invited to take part in the study, who filled in the pre-survey, the time-use diary and the post-survey in each wave

	**Wave 1** **March 2018**	**Wave 2** **October 2018**	**Wave 3** **March 2019**	**Wave 4** **October 2019**
Invited	61	60	59	56
Pre-diary survey	60	56	55	49
Time-use diary	51	47	49	42
Post-diary survey	51	53	54	48
Cases used in the analyses	54	50	51	45

For the analyses, we use the sample of respondents with a valid response for (at least one of) the dependent variables (total *n* = 60, not all observed in every wave, 200 observations over the waves, see numbers in Table 1). The percentage of missing values in this sample varies by variable (see Table A1 in the appendix) and missings were imputed (see also further for the method of imputation).

### Experimental and control groups

The impact of the shorter workweek can be studied by comparing the well-being scores in waves 3 and 4 (post-measurements) to their scores in wave 1 and wave 2 (pre-measurements). In addition, changes in well-being can be compared between what we might call experimental and control groups. The trial was not designed to have a randomly assigned control group to compare the experimental group with. Yet when 30 h became the new maximum number of work hours, some workers’ work time was reduced to a greater extent than others’. Thus, intervention effects can be expected to depend on workers’ initial work hours (as a proxy for the amount of change in work hours), as is explained in the variables section on work hours.

### Variables

Table 2 shows the mean values and percentages of all dependent and independent variables for each wave.

**Table 2:** Mean values and percentages of all dependent and independent variables for every wave

	**Wave 1**	**Wave 2**	**Wave 3**	**Wave 4**
	***N* = 54**	***N* = 50**	***N* = 51**	***N* = 45**
**Dependent and explanatory variables**	*Mean*	*Mean*	*Mean*	*Mean*
Work-family conflict (-)	3.6	3.1^**^	2.2^**^	2.6
Positive work experience ( +)	7.7	7.6	7.4	6.5^***^
General well-being ( +)		6.2	6.3	6.0
Sufficient free time ( +)	4.1	4.0	4.7^***^	4.5
Duration leisure time	2.7	2.7	2.6	2.7
Duration social participation	1.3	1.6	1.6	1.5
Duration housework	2.0	2.2	2.5^**^	2.3
Household stress (-)	3.4	3.3^*^	3.1	3.2
Satisfaction division housework ( +)	3.7	3.7	3.7	3.5
Schedule control ( +)	4.0	4.0	4.0	4.0
Satisfaction work pressure ( +)	3.4	3.4	3.6 *	3.6
**Working hours groups**	*%*	*%*	*%*	*%*
36-h group	42.6%	42.0%	41.2%	48.9%
28 to 34 h group	27.8%	30.0%	31.4%	31.1%
-26 h group	29.6%	28.0%	27.5%	20.0%
Work hours increased	1.9%	2.0%	3.9%	4.4%
**Control variables**	*%*	*%*	*%*	*%*
Living with a child	33.3%	36.0%	37.3%	40.0%
Living with a partner	81.5%	80.0%	88.2%	86.7%
Transitioning teams	27.8%	26.0%	31.4%	33.3%

( +) positively formulated (-) negatively formulated

Results pooled from 10 imputed samples. *p =  < 0.1; **p =  < 0.05; ***p =  < *0.01* for change between subsequent waves (i.e. comparison with previous wave) using pairwise t-tests for dependent samples (see also Table A4 in the Appendix for the exact differences and *p*-values)

### Dependent variables

To address the multidimensional character of well-being, workers’ well-being was assessed in three domains: work-family well-being, job-related well-being, and general (or context-free) well-being.

*Work-family conflict* is used as a measure of the work-family well-being dimension. The work-family conflict scale is constructed as a sum score (rescaled to 0–10) of four items scored on a 4-point Likert-scale (ranging from 1 never or almost never to 4 always or almost always) with a Cronbachs α > 0.79. Items include [How often does it happen that…] ‘you feel less involved with your family/friends because of the requirements of your work?’ or ‘you feel that you lag behind the events at home?’. The higher the score, the higher the conflict experienced.

*Positive work experience* is the variable that we use as a proxy for job-related well-being. The scale measures the pleasure, meaningfulness and challenge in work and is a sum scale (rescaled to 0–10) based on five items scored on a 4-point Likert-scale (ranging from 1 never or almost never to 4 always or almost always) (Cronbachs α > 0.88). Items include ‘I find it pleasant to start the workday’ and ‘I love the challenge in my work’ (see appendix for full list). The higher the score on the scale, the better the subject’s work experience.

*General (context-free) well-being* was measured using 5 items from the short version of the Warwick-Edinburgh Mental Well-being Scale (SWEMWBS), all measured on a 5-point Likert scale ranging from never to always (Cronbachs α > 0.74) to construct a general well-being sum score (rescaled to 0–10). SWEMWBS represents mostly aspects of psychological and eudemonic well-being (as part of the full 14-item WEMWBS; [40]). Even though SWEMWBS construct validity was confirmed in diverse populations (e.g., [13, 44]), in our study (longitudinal) confirmatory factor analyses led us to exclude two items (which improved model fit from poor to fair as *RMSEA* changed from 0.125 to 0.077 and *CFI* from 0.729 to 0.932), which also improved longitudinal measurement invariance (see further). Items include [in the last two weeks] ‘I’ve been feeling optimistic about the future’ and ‘I’ve been feeling relaxed’. Whereas the other measures were administered in each of the four waves, the SWEMWBS scale was only included in the surveys starting in wave 2.

### Work hours

**Fig. 1:**
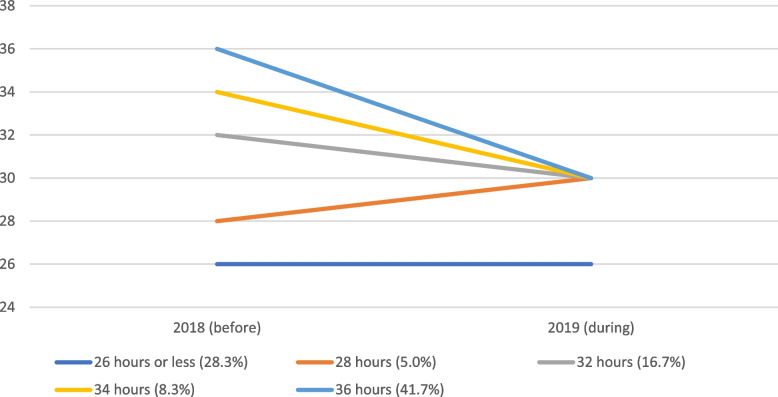
Reduction (or increase) of working time for different working time groups between 2018 (before the trial) and 2019 (during the trial) (research sample *N* = 60, same as used in further analyses)

In this study, employees were categorized into three groups: those working 26 h or less before the start of the trial (i.e., part-time employees), those working 28 to 34 h, and those working 36 h before the trial. The 36-h group makes up 41% to 49% of employees in our data, with exact numbers varying over the different waves. The 28–34-h group represents 28% to 31% over the waves and the 26-h or less group is between 20 and 30%. The fluctuations in numbers are due to non-response and the imposition of strict quality checks on the time diaries, as well as changes in the composition of employees at Femma Wereldvrouwen over time as some employees retired, resigned or new ones were hired. As stated above, all full-time employees (working 32, 34 or 36 h) reduced their working hours to 30 per week in 2019. The 36-h group is the most interesting here as they experienced the largest reduction in work time (6 h per week; see also Fig. 1). The 28 to 34 h group consists of employees with a smaller reduction in work time (4 or 2 h per week). A few employees even increased their work time (voluntarily, from 28 to 30 h), which we control for with a dummy variable (1 = increased work hours, 0 = not increased work hours). The part-time group that worked 26 h or less per week did not reduce their weekly working hours and as such can be considered as some sort of control group for the full-time workers. This group, similar to the 28–34 h group, differs from the 36-h intervention group as the workers are older, do not have children at home, and experienced less work-family conflict and household stress at baseline (see Table A3 in the appendix). As discussed further in this section, we control for these socio-demographic background characteristics as well as for (time-varying) work and private life-related variables, which is in line with JRC’s recommendations [10]. Regarding the effect of work time reduction, we expect to find the largest changes between waves among the 36-h group, followed by smaller changes in the 28–34 h group, and no effects in the 26-h or less group.

### Time-changing mediating variables

#### Time-use variables

Most of the time-use-related variables are derived from the 7-day time diaries respondents kept and measured based on the total duration of time spent on activities during these 7 days (and rescaled to number of hours per day). Only the variable ‘sufficient free time’ is derived from the pre-diary survey.

*Sufficient free time* was measured by the question ‘To what extent do you feel you have sufficient free time?’, scored from 1 (too little) to 7 (more than enough). This measures the adequacy of free time experienced by the respondent.

*Time spent on leisure* activities (hobbies, games, TV and video, cultural participation, going out, sports, recreation, music, reading).

*Time spent on social participation* (talking, visiting family, friends, …).

*Time spent on household work* (cleaning, cooking, washing, …).

#### Work variables

*Schedule control* is a scale score calculated as the average on three items scored on a 5-point Likert-scale (ranging from 1 totally disagree to 5 totally agree). Items include: ‘I can decide for myself how I do my work’; ‘I can decide for myself how much work I do in a day’; ‘I can decide for myself what work I do in a day’.

*Satisfaction with work pressure* was measured by one item ‘Please indicate the extent to which you are currently satisfied with the following aspects of your work: work pressure’ on a 5-point Likert-scale (ranging from 1 not at all satisfied to 5 very satisfied).

#### Household variables

*Household stress* is a scale score calculated as the average of four items on a 5-point Likert-scale (ranging from 1 totally disagree to 5 totally agree). Items include ‘There are moments when I am short of hands in the household’; ‘I feel stressed when I think about the household tasks that still have to be done’; ‘I often postpone my household chores’; ‘The time for household work is planned and fixed in advance’ (reverse coded).

*Satisfaction with division of housework* is based on the question ‘To what extent are you satisfied with the division of housework between you and your partner?’. The question was answered with a 5-point Likert-scale (ranging from 1 not at all satisfied to 5 very satisfied).

### Control variables

#### Socio-demographic variables

*Age* of the respondent was measured based on their age in 2020. In our analyses, we centered the age variable at its mean (48). The mean age of the 36-h group in 2020 was 38,5. This is the youngest group. The mean age of the 28 to 34-h group was 54 and the mean age of the 26-h or less group was 58,2.

*Living with children:* We also controlled for the composition of the household, taking into account whether the respondent lived with children under 18 in their household or not (reference category). With this, we want to control for the extra care responsibilities in the household.

*Living with partner:* We discern those who live with their partner (reference category) from those who do not live with their partner or do not have a partner.

#### Work context

*Transitioning teams:* We control for membership of a teams that had difficulties dealing with the organizational changes and reorganization (see the context section). Being a member of a team that did not experience particular adjustment problems acts as the reference category.

Missing values were imputed using multiple imputations by chained equations (MICE), on the full set of the analyses variables, including the dependent variables, adapted to the measurement level of the variables, and using the original Likert items to derive (passively) imputed scale scores. We generated 10 complete datasets. Results of the repeated complete data analyses were combined by averaging estimates and with adjusted standard errors using Rubin’s rules (using the R-libraries *mice, broom.mixed* and *mitml*).

### Method

Longitudinal measurement invariance of the Likert-item composed scale variables was checked (using *sem* and *Lavaan* libraries in R) to assess the psychometric equivalence of the constructs for individuals over time. The procedure is to specify confirmatory factor analysis structural models for each wave simultaneously (and allowing covariance over time both in latent constructs and in corresponding items) and compare this model with models with additional constraints to test measurement invariance over time (e.g. [36]). Full scalar measurement invariance was reached for schedule control, household stress and positive work experience (see also Table A2 in the appendix). For work-family conflict and the well-being scale, metric invariance is confirmed. Scalar measurement invariance can be accepted according to certain model fit criteria (i.e., model with the lowest BIC or change in RMSEA < 0.01), even though it would be rejected by the (overly strict) likelihood ratio model comparison tests (respectively 21, *df* 9, *p* = 0,012, and 169, *df* = 8, *p* = 0.031).

To analyze the effects of work time reduction, we model change over time (waves) using multilevel growth models (cf. [39]), with measurements at each of the waves as first-level observations, nested within the respondents as the second-level grouping variable. For the two context-specific well-being measures (work-family conflict and positive work experience), we specify a random slope (for wave) model, for the general well-being measure, we specify a random intercept model (as we only have three measurement occasions). In all these models, the wave is included as a predictor using dummy coding (reference: first wave), as well as the work hours group (36-h group as reference group), and the interaction terms for these two variables which allow detecting different changes over time by work hours group. These multilevel analyses were performed in R using the *lme4* library. The number of observations in our sample exceeds the minimum recommendation by Hox and McNeish [23] for longitudinal samples to obtain adequate parameters from hierarchical linear modeling when using the REML method.^1^

For each of the three well-being measures, we report on two models. Model 1 is the base model with the three working time groups, the four waves and their interaction terms, including controls for socio-demographic background and work context. In Model 2 we add the time-changing mediating variables to the Model 1 specification. Here we are interested in a) how concomitant changes in work and home/private circumstances might explain work time reduction effects and b) which of these changes are related to changes over time in well-being (longitudinal rather than cross-sectional effect estimations). By including workers’ initial levels in these time-changing characteristics, we control for person-level (between) differences and ensure that the effect parameters for the time changing work and household characteristics can be interpreted as the effects of changes over time (within differences). Finally, for general well-being we report on a third model, in which the time-changing variables for work-family conflict and positive work experience (as well as workers’ initial levels as control variables) are included as well.

## Findings

**Fig. 2:**
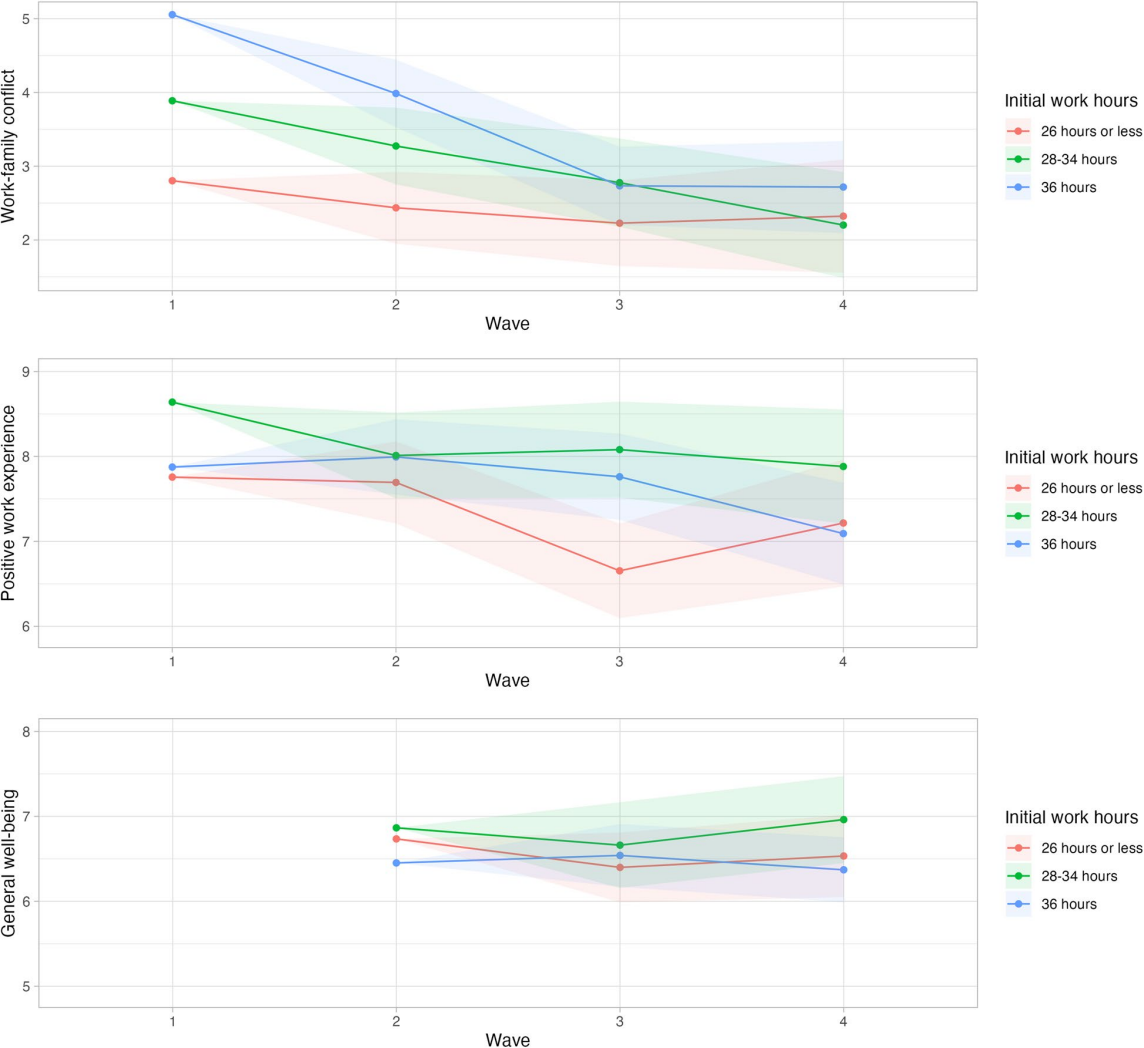
Growth curves for the three work hour groups of the three tested domains of well-being: work-family conflict, positive work experience and general well-being (based on the Model 1 parameters reported in Table 3) *Note:* Estimated means calculated at age 38, living with a partner and having children at home. Error bands represent the standard error of the estimated wave effects per work hours group with reference to the first wave (second wave for general well-being). To highlight the differences, the Y-axes in the figures do not cover the complete range. This scaling visually accentuates the differences between groups, which are not necessarily statistically significant

Table 3 reports on the results from the multilevel growth models for the three well-being measures. To facilitate interpretation, in Fig. 2 we also visualize the growth curves for the three work hour groups, based on the estimates from Model 1 in Table 3. These figures visualize the model-based estimated means for each work hour group in all waves. Apart from illustrating the differences between the groups, the figures show the changes over time for each of the work hour groups. The error bands illustrate the precision of estimated within-individual change and depict the standard error of the estimated wave effects in relation to the first wave.

**Table 3:** Effect parameters (unstandardized B’s) and significance levels for the three tested domains of well-being: work-family conflict, positive work experience and general well-being

	**Work-family conflict**		**Positive work experience**		**General well-being**		
	**Model 1**	**Model 2**	**Model 1**	**Model 2**	**Model 1**	**Model 2**	**Model 3**
**Time (ref = wave 1, 36 h group)**							
Wave2	-1.07^**^	-0.99^**^	0.12	-0.06			
Wave3	-2.32^***^	-1.66^***^	-0.11	-0.49	0.09	-0.39	-0.38
Wave4	-2.34^***^	-1.68^***^	-0.78	-1.23^**^	-0.08	-0.35	-0.24
**Control group effects**							
*Working hours groups (ref* = *36 h group, wave 1)*							
-26 h group	-2.25^**^	-1.32^*^	-0.12	-0.14	0.28	0.09	-0.42
28 to 34 h group	-1.17	-1.35^*^	0.76	0.77	0.41	0.37	-0.16
-26 h group * wave 2	0.70	0.44	-0.18	0.25			
-26 h group * wave 3	1.75^**^	0.92	-0.99	-0.33	-0.42	-0.01	0.14
-26 h group * wave 4	1.86^**^	1.42	0.24	0.74	-0.12	0.18	0.17
28 to 34 h group * wave 2	0.46	0.14	-0.75	-0.37			
28 to 34 h group * wave 3	1.21	0.79	-0.44	0.02	-0.29	-0.07	-0.03
28 to 34 h group * wave 4	0.65	0.50	0.02	0.45	0.18	-0.08	-0.04
**Explanatory variables—time changing**							
Sufficient free time		-0.36^***^		0.07		0.27^**^	0.22
Duration leisure time		-0.14		0.05		0.11	0.12
Duration social participation		0.00		-0.00		0.05	0.06
Duration housework		-0.04		-0.23		-0.13	-0.02
Household stress		-0.07		-0.45		-0.35	-0.31
Satisfaction division housework		0.04		-0.06		0.28^*^	0.23
Schedule control		-0.76^***^		0.77^***^		0.22	0.08
Satisfaction work pressure		-0.37^**^		0.04		0.03	0.03
Work-family conflict							0.001
Positive work experience							0.14^*^
**Explanatory variables—start position (control variables)**							
Sufficient free time—start		-0.37^**^		-0.15		-0.17	-0.05
Duration leisure time—start		0.05		-0.04		-0.11	0.01
Duration social participation—start		-0.06		-0.15		-0.04	0.09
Duration housework—start		0.23		-0.05		0.54^**^	0.60^***^
Household stress—start		0.05		0.25		0.04	0.20
Satisfaction division housework—start		-0.18		0.16		-0.28	-0.18
Schedule control—start		1.55^***^		-0.90^*^		-0.03	0.01
Satisfaction work pressure—start		-0.35^*^		0.56^**^		0.40^*^	0.11
Work-family conflict—start							-0.03
Positive work experience—start							0.15
**Socio-demographic control variables**							
Age	0.01	0.01	0.03	0.04	-0.02	-0.03	-0.02
Living with a child (ref = without child)	0.78	-0.06	1.03^*^	1.49^**^	0.19	0.68	0.42
Not living with partner (ref = with partner)	-0.30	0.09	-0.38	-0.13	-0.85^*^	-0.58	-0.68
**Other control variables**							
Transitioning team (ref = teams that did not struggle)	-0.17	-0.49	0.60	0.72	-0.10	0.06	0.09
Transitioning team * wave 2	0.50	0.82	-0.00	-0.24			
Transitioning team * wave 3	0.35	0.43	0.39	0.43	0.62	0.80	0.66
Transitioning team * wave 4	1.90^**^	1.33^*^	-1.75^**^	-1.34^*^	-0.50	-0.25	-0.17
Observations	200	200	200	200	146	146	146
Respondents	60	60	60	60	58	58	58
Log Likelihood	-373	-355	-370	-367	-235	-233	-232
AIC	799	781	794	820	511	537	545
BIC	888	896	883	962	570	645	664

All analyses controlled for increasing work hours (and interaction with wave)

^*^p =  < *0.1,* ^**^p =  < *0.05,* ^***^p =  < *0.01*

### Work-family conflict

Work-family conflict decreased significantly over the waves for the 36-h group (Model 1 in Table 3, see also Fig. 2). Already in wave 2 (October 2018) when the shorter workweek trial had not even started, work-family conflict decreased (B = –1.07; 95% confidence interval: [–1.97, –0.17]) among the 36-h group. This decrease becomes even larger during the 30-h workweek trial in wave 3 (B = –2.32; [–3.36, –1.28]) and in wave 4 (B = –2.34; [–3.56, –1.12]), which is in line with hypothesis H1a. Among the less than 26-h group, this decrease in work-family conflict is much smaller as the time effects differ significantly from those in the 36-h group, (if summed, only small negative effect parameters remain over time for the less than 26-h group, and when used as the reference group in analyses not shown here we did not find significant changes over time). As mentioned in the data section, this group did experience a lower work-family conflict to start with (cf. main effect for work hours group), which is probably explained by their standard shorter working hours (before the experiment). The time effects for the in-between 28 to 34-h group do not differ significantly from those of the 36-h group, but the (positive) effect parameters for the time interaction terms (in combination) indicate that the decrease in work-family conflict over time for the 28–34 h group is smaller than the 36-h group’s and larger than the less than 26-h group’s. Those who reduced their working hours more thus reap the most benefits in work-family conflict. This beneficial effect of work time reduction, however, is much less clear for the members of the few teams that had difficulty in coping with the organizational changes. In particular, in wave four, work-family conflict started to increase among the (remaining) employees of these teams. The negative work-family effects for these team members were probably caused by the spill-over of negative work experiences (see further).

Model 2 allows us to look into the effects of concomitant changes in work and the private realm. Holding these time-changing characteristics constant, the decrease in work-family conflict over time for the 36-h group remains but is less pronounced. Slightly less than 30% of the decrease in work-family conflict during the shorter workweek (wave 3 and 4) among the 36-h group is explained by concomitant changes in work and private circumstances (e.g., B of –2.32 becomes –1.66 in wave 3; –1.66/–2.32 = 71.6% of the effect remains). The main changes over time that are related to changes in work-family conflict concern schedule control, satisfaction with work pressure and sufficient free time. Increases over time in schedule control, satisfaction with work pressure and sufficiency of free time, correlate with a decrease in work-family conflict. For hypothesis H1b we expected that if extra time was spent on leisure time this would impact work-family conflict. Yet we only find the subjective sufficiency of free time experienced by the employees, in addition to two work-related variables, to play a role in explaining some of the reduction in conflict between work and family.

### Positive work experience

Although there is a negative tendency in all work hour groups (in particular in wave 4, and most strongly for members of the teams who struggled with the organizational changes), no significant general change is apparent in positive work experience over the waves (Model 1 in Table 3, see also Fig. 2). This does not provide support for hypothesis H2a. We even see that among employees who were part of a team that had difficulties with the reorganizational changes, the positive work experience decreased strongly (and significantly) in wave 4 (October 2019) in comparison with wave 1 (March 2018) (B = –1.75; [-3.31, -0.19]).

Holding constant the time-changing work and home variables (Model 2), positive work experience does decrease strongly and significantly for employees in the 36-h group in wave 4 compared to wave 1 (B = –1.23; [-2.45, -0.01]). Nine months into the shorter workweek, these employees would have experienced a less positive work environment/experience but this negative effect is offset (in part) by some other (positive) concomitant changes. The most important time-changing variable affecting change in positive work experience is schedule control: an increase in schedule control leads to an increase in positive work experience (controlled for baseline differences). Schedule control acts as a suppressor variable: if it had not changed over the waves, the 36-h group would have had a lower positive work experience (B = –1.23). Schedule control can thus help alleviate negative experiences at work. This partly supports hypothesis H2b in which we expected (changes in) schedule control to affect job-related well-being.

### General well-being

For general well-being, we only have data for the three last waves, so October 2018 (three months before the start of the trial) is the reference wave. There is no significant change in general well-being over the waves, for either of the three groups (Model 1 in Table 3, see also Fig. 2), so hypothesis H3a is not confirmed. Holding the time-changing characteristics of work and home contexts constant (Model 2), general well-being tends to decrease over time, which is partly due to the decreased work enjoyment, particularly in wave 4 (Model 3), although effects are not statistically significant.

Among the changes in work and private context, having sufficient free time is an important predictor for general well-being. Increases over time in the experience of sufficient free time and satisfaction with the division of housework at home are related to increases in general well-being (B = 0.27 [0.01, 0.53]; B = 0.28 [-0.01, 0.57]). As positive work experience and work-family conflict might affect general (or context-free) well-being, we added these to Model 3. Indeed, a change in positive work experience impacts general well-being, more specifically an increase in positive work experience over time is related to improvements in general well-being (B = 0.14 [-0.03, 0.31]), partly supporting hypothesis H3b. The decrease in positive work experience however offset part of the potential positive effects of work-time reduction for general well-being. Work-family conflict, on the other hand, does not appear to be related to general well-being longitudinally.

## Discussion and conclusion

In this quasi-experiment, the shorter workweek in itself did not seem to significantly impact general well-being. After nine months, employees whose weekly work hours were reduced with 6 h experienced lower job-related well-being (positive work experience), in particular if the increase in schedule control for this group was accounted for. This corresponds to some of the findings from the national working time reduction in Korea [37], yet is unlike findings from many other small-scale experiments [2, 18, 42, 46] and from national implementations in France and Portugal [31] where job and life satisfaction did increase during the shorter workweek. However, we did find an improvement in work-family conflict over the waves for the women whose working hours were reduced. As of wave 2, in October 2018, the conflict between work and family started to decrease, even more so during the shorter workweek. This improvement was partly explained by increased schedule control, satisfaction with work pressure and perception of sufficient free time over time. When the shorter workweek led to more of these, work-family conflict decreased. Yet also without changes in these work and family context characteristics, the shorter workweek decreased the experienced work-family conflict. That we find an impact on work-family conflict could have been expected as a shorter workweek reduces the time spent on work and releases time for private pursuits such as family. This was also an important aim of the 30-h workweek at Femma Wereldvrouwen. Especially for women who often bear more responsibility over household work and care tasks, such a reduction might be beneficial [1, 6, 22, 49]. However, in the case of this trial, not only the time spent at work was reduced, but the organization also put in efforts to decrease work pressure by outsourcing work and hiring new employees to take over tasks that could no longer be performed by the former employees due to reduced work time in the shorter workweek. This had a positive impact on work-family conflict (already at wave 2). Other research has shown that having to do the same work in a shorter workweek can lead to higher work intensity and increase stress among employees [11, 29, 37].

For both work-family conflict and positive work experience change over time in schedule control has a beneficial impact. Improving schedule control can thus be a good strategy to alleviate possible negative impacts of a shorter workweek (such as changes in teams or the organization of work) or increase positive impacts even more. This relates to Anttila’s finding [2] that the loss of some schedule control in the 6-h workday experiments in municipalities in Sweden led to some adverse effects concerning work and family.

Context is important to understand shorter workweek trials as they do not take place in a vacuum but in the real world. The same reorganization that helped outsource work to not increase work pressure also installed new self-managing teams four to three months before the start of the shorter workweek trial. Self-management is not easy for everyone and some teams did not handle it very well. In the middle of 2019, management had to intervene in these teams and some employees that could no longer ground in the new organizational set-up were laid off or decided to leave the organization themselves. Through in-depth interviews that were part of the broader research project, we know that this negatively impacted the atmosphere in some of the teams. This contextual info might help understand the findings for job-related well-being (and spill-over into general well-being) for the few teams that had a harder time. It also shows that a collective decrease in working time should go hand in hand with a careful rethinking of work practices, and this should be an ongoing concern. Allowing enough time for interventions such as new self-managing teams to settle before introducing other interventions is an important lesson as well. Femma Wereldvrouwen learned that the time between the reorganization and the introduction of the shorter workweek was too short as teams needed time to settle in their new ways of working. Both during and after the trial, Femma Wereldvrouwen made some adjustments in the teams such as merging some of the teams and reintroducing bilateral coaching.

We did not find any significant effects of any of our time use variables based on the 7-day diary. We had expected that increases in leisure time in the shorter workweek might have positively affected some well-being indicators. The only time-use-related variable that seemed to matter was the experience of sufficient free time. This variable is a subjective evaluation (originated from the surveys), which correlates significantly but weakly with time spent on leisure time (from the diaries) (*r* = 0.265). The dependent variables are also subjective measures, and originate from the surveys as well. It could be a common method effect that these subjective measures correlate more strongly with each other than with the durations of time spent on activities from a random week, which are more objective measures. Additionally, the reference period of the dependent variables’ items is two weeks or ‘in general’ and thus differs from the reference period of the diary, which is 7 days.

The trial lasted one year, which is as long or longer than other recent trials with reduced work hours (e.g. [11, 42]). For work-family conflict, we see the start of the positive impact of a shorter workweek already in wave 2, three months before the start of the trial. This might point to some sort of Hawthorne effect, where the fact of being part of a trial, here the fact of working for an employer that is involved with workers’ well-being and that will trial a shorter workweek (and concomitant preparations), in itself already impacts the experienced well-being of employees. Schor [38] mentioned a similar finding for job satisfaction in the recent experiments with the four-day workweek in the US. Lastly, the trial was limited to one organization, where partners of employees did not reduce working hours. Within the vision of Femma Wereldvrouwen, the aimed result of a better and fairer combination of paid and unpaid work and gender equality would only be attained if everyone worked less. In this case, it was only one organization with mostly female employees. For now, the shorter workweek at Femma Wereldvrouwen did improve women’s experiences of their work-family combination, which is an important step towards more gender equality as women still bear most of the burden of the double shift.

Despite the limitations of the non-randomized study design and the small sample of highly-educated female knowledge workers, our longitudinal design with two pre-measurements and two post-intervention-measurements including time-use data, as well as intervention and some sort of control groups, provided us with a unique dataset and broad set of measures to evaluate the impact of work time reduction. Although the results from this trial might not transfer to other contexts, it does provide compelling evidence that shorter workweeks can potentially lead to significant improvements in work-life balance, i.e. through reduced work-family conflict as evidenced in our longitudinal analysis. Although the other well-being impacts were variable, the marked benefits for female employees underscore the potential for tailored work time reductions to enhance the combination of work and family life. Our study can inform other trials, and organizations considering trialing a shorter workweek and furthers the limited scientific knowledge on the topic.

### Supplementary Information

Supplementary Material 1.

## Data Availability

The raw data on the employees of the organization that trialled the shorter workweek as well as the aggregated data are protected and shared only with researchers within our research group. The organization is very small and anonymity cannot be guaranteed. The organization only consented on the data being used by us. Upon request, our syntaxes of analyses can be made available. Please e-mail Francisca.Mullens@vub.be.

## Footnotes

1 The use of small sample sizes is not uncommon in longitudinal studies. We analyze LGM within the HLM framework. Even in the case of small numbers, HLM seems to be a viable method of analysis with good performance to recover parameters and providing the least-biased method to account for clustering [33, 50]. The number of respondents in our sample seems adequate, in particular as we use the REML method (instead of ML), whose performance is better for smaller samples. E.g. based on a review of studies, McNeish & Stapleton [34] recommend in the case of a continuous outcome a minimum of 15 to 30 clusters for unbiased fixed effects parameters and of at least 30 clusters for unbiased fixed effects standard errors. For longitudinal samples, Hox and McNeish [23] recommend a minimum of 20 clusters for adequate fixed effect parameters when using REML.
