# Supplementary material for: An organizational working time reduction and its impact on three domains of mental well-being of employees: a panel study
Source: BMC Public Health. 2024 Jun 28;24:1727. doi: 10.1186/s12889-024-19161-x (PMC11212234; doi:10.1186/s12889-024-19161-x)
Supplement: Supplementary file 1 [file 12889_2024_19161_MOESM1_ESM.docx]

Appendix 

Table A1 Items, source, Cronbachs α, range and % missings for all dependent and independent variables in the analyses

| Scale/variable | Items | Source | Cronbachs α | Range (min-max) | Missings |
| --- | --- | --- | --- | --- | --- |
| Work-family conflict |  | post-diary survey | 0.798 | 0-10 | 12.5% |
|  | How often does it happen that… |  |  |  |  |
|  | you feel less involved with your family/friends because of the requirements of your work? |  |  | 1-4 | 8.5% |
|  | you feel that you lag behind the events at home? |  |  | 1-4 | 7.5% |
|  | you have so much work to do that you do not have time for your hobbies? |  |  | 1-4 | 7.5% |
|  | the requirements of your work make it hard to relax? |  |  | 1-4 | 5.5% |
| Positive work experience |  | pre-diary survey | 0.884 | 0-10 | 3.0% |
|  | I find it pleasant to start the workday |  |  | 1-4 | 0.0% |
|  | I still find my work fascinating, every day again |  |  | 1-4 | 0.5% |
|  | I have fun working |  |  | 1-4 | 0.0% |
|  | I love the challenge in my work |  |  | 1-4 | 2.0% |
|  | I have the feeling that my work is meaningful |  |  | 1-4 | 1.0% |
| General well-being |  | post-diary survey | 0.742 | 0-10 | 6.8% |
|  | Please select the option that best describes your experience of each over the last 2 weeks |  |  |  |  |
|  | I've been feeling optimistic about the future |  |  | 1-5 | 5.5% |
|  | I've been feeling relaxed |  |  | 1-5 | 5.5% |
|  | I've been dealing with problems well |  |  | 1-5 | 4.8% |
|  | I've been thinking clearly |  |  | 1-5 | 4.8% |
|  | I've been able to make up my own mind about things |  |  | 1-5 | 5.5% |
| Sufficient free time | To what extent do you feel you have sufficient free time ? | pre-diary survey |  | 1-7 | 3.0% |
| Duration leisure time | hours/week | diary |  | 0-6.4 | 6.5% |
| Duration social participation | hours/week | diary |  | 0-5.5 | 6.5% |
| Duration housework | hours/week | diary |  | 0-6.9 | 6.5% |
| Household stress |  | pre-diary survey | 0.744 |  | 3.5% |
|  | There are moments that I am short of hands in the household |  |  | 1-5 | 2.5% |
|  | I feel stressed when I think of all the household chores that still have to be done |  |  | 1-5 | 1.5% |
|  | I often postpone my household chores |  |  | 1-5 | 2.0% |
|  | The time for the household work is planned and fixed in advance (reverse coded) |  |  | 1-5 | 1.0% |
| Satisfaction division housework | To what extent are you satisfied with the division of housework between you and you partner? | post-diary survey |  | 1-5 | 20.0% |
| Schedule control |  | pre-diary survey | 0.807 |  | 1.0% |
|  | I can decide for myself how I do my work |  |  | 1-5 | 0.5% |
|  | I can decide for myself how much work I do in a day |  | 1-5 | 0.5% |
|  | I can decide for myself what work I do in a day |  |  | 1-5 | 1.0% |
| Satisfaction work pressure | Please indicate the extent to which you are currently satisfied with the following aspects of your work: work pressure | pre-diary survey |  | 1-5 | 1.0% |

Table A2: Measurement invariance analyses of the dependent variables

|  | Model fit parameters |  |  |  |  | Model comparisons |  |
| --- | --- | --- | --- | --- | --- | --- | --- |
|  | 𝜒² | df | p | RMSEA | CFI | BIC | Δ𝜒² | Δdf | p |
| Work-family conflict |  |  |  |  |  |  |  |  |  |
| configural invariance | 134.4 | 74 | .000 | .117 | .853 | 1.668 | - | - | - |
| metric invariance | 144.9 | 83 | .000 | .112 | .850 | 1.642 | 10.5 | 9 | 0.312 |
| scalar invariance | 166.1 | 92 | .000 | .116 | .820 | 1.626 | 21.2 | 9 | 0.012 |
| Positive work experience |  |  |  |  |  |  |  |  |  |
| configural invariance | 172.1 | 134 | .015 | .069 | .948 | 1.912 | - | - | - |
| metric invariance | 186.4 | 146 | .013 | .068 | .944 | 1.877 | 14.4 | 12 | 0.275 |
| scalar invariance | 201.0 | 158 | .012 | .067 | .941 | 1.842 | 14.5 | 12 | 0.271 |
| General well-being |  |  |  |  |  |  |  |  |  |
| configural invariance | 95.7 | 72 | .032 | .077 | .932 | 1.417 | - | - | - |
| metric invariance | 105.6 | 80 | .029 | .076 | .926 | 1.395 | 98.2 | 8 | 0.278 |
| scalar invariance | 122.4 | 88 | .009 | .084 | .901 | 1.380 | 168.8 | 8 | 0.031 |

Table A3. Baseline differences between work hours group in mean values and percentages of all dependent and independent variables (wave 1, N=54)

|  | 36-hours | 28-34 hours | 26 or less hours |
| --- | --- | --- | --- |
|  | N=23 | N=15 | N=16 |
| Dependent variables | Mean (=ref.) | B | p | B | p |
| Work-family conflict (-) | 4.7 | -2.4 | 0.003 | -1.5 | 0.060 |
| Positive work experience (+) | 7.7 | -0.2 | 0.704 | 0.4 | 0.460 |
| General well-being (+) (*) | 6.2 | 0.0 | 0.928 | -0.3 | 0.577 |
| Explanatory variables |  |  |  |  |  |
| Sufficient free time (+) | 3.5 | 1.5 | 0.002 | 0.7 | 0.184 |
| Duration leisure time | 2.6 | 0.1 | 0.770 | 0.3 | 0.423 |
| Duration social participation | 1.1 | 0.6 | 0.068 | 0.2 | 0.545 |
| Duration housework | 1.5 | 1.0 | 0.001 | 0.6 | 0.048 |
| Household stress (-) | 3.9 | -0.9 | 0.001 | -0.7 | 0.006 |
| Satisfaction division housework (+) | 3.7 | -0.2 | 0.653 | 0.2 | 0.664 |
| Schedule control (+) | 4.1 | -0.2 | 0.101 | -0.2 | 0.175 |
| Satisfaction work pressure (+) | 3.2 | 0.6 | 0.052 | -0.1 | 0.694 |
| Control variables |  |  |  |  |  |
| Age | 37.9 | 21.0 | 0.000 | 16.8 | 0.000 |
| Living with a child (proportion) | 0.70 | -0.57 | 0.000 | -0.70 | 0.000 |
| Living with a partner (proportion) | 0.78 | 0.22 | 0.083 | -0.12 | 0.359 |
| Transitioning teams (proportion) | 0.35 | -0.16 | 0.285 | -0.08 | 0.594 |

Note: (+) positively formulated (-) negatively formulated: (*) reported for wave 2 as this measure is not available for wave 1 (N=50).

Coefficient estimates (B) and p-values from t-tests of the coefficients estimates (p), pooled from linear regression models applied to 10 imputed samples.

Table A.4. Change between waves in time-changing variables

|  | From wave 1  to wave 2 | From wave 2  to wave 3 | From wave 3  to wave 4 |
| --- | --- | --- | --- |
|  | N=48 | N=43 | N=42 |
|  | difference | p value | difference | p value | difference | p value |
| Work-family conflict (-) | -0.69 | 0.011 | -0.71 | 0.011 | 0.30 | 0.363 |
| Positive work experience (+) | -0.11 | 0.685 | -0.34 | 0.141 | -0.94 | 0.007 |
| General well-being (+) | 0.14 | 0.516 | 0.11 | 0.565 | -0.36 | 0.143 |
| Sufficient free time (+) | 0.02 | 0.901 | 0.71 | 0.001 | -0.15 | 0.494 |
| Duration leisure time | 0.05 | 0.748 | 0.14 | 0.392 | 0.11 | 0.580 |
| Duration social participation | 0.20 | 0.296 | 0.15 | 0.289 | -0.02 | 0.903 |
| Duration housework | 0.12 | 0.407 | 0.30 | 0.016 | -0.09 | 0.580 |
| Household stress (-) | -0.14 | 0.071 | -0.15 | 0.146 | 0.04 | 0.647 |
| Satisfaction division housework (+) | 0.10 | 0.560 | 0.01 | 0.979 | -0.19 | 0.337 |
| Schedule control (+) | 0.07 | 0.328 | -0.06 | 0.539 | -0.01 | 0.961 |
| Satisfaction work pressure (+) | 0.09 | 0.473 | 0.24 | 0.081 | 0.00 | 1.000 |
| Living with a child | 0.00 | - | 0.00 | - | 0.00 | - |
| Living with a partner | -0.02 | 0.323 | 0.02 | 0.323 | 0.00 | - |
| Transitioning team s | 0.02 | 0.323 | 0.00 | - | -0.02 | 0.323 |

Note: (+) positively formulated (-) negatively formulated 

Mean difference and p-values from pairwise t-tests for dependent samples applied to 10 imputed samples, using multiple imputation t-test (using R-project library Mkinfer).
